# Reconsidering the spiritual–existential dimensions of perceived burden in advanced cancer

**DOI:** 10.1017/S147895152610234X

**Published:** 2026-04-24

**Authors:** Ishlakhatus Sa'idah, Najlatun Naqiyah, Titin Indah Pratiwi, Budi Purwoko, Bakhrudin All Habsy, Moh. Ziyadul Haq Annajih, Diana Vidya Fakhriyani

**Affiliations:** 1 Guidance and Counseling Department, Universitas Negeri Surabaya, Indonesia; 2 Islamic Guidance and Counseling Department, Institut Agama Islam Miftahul Ulum Pamekasan, Indonesia; 3 Guidance and Counseling Department, Institut Agama Islam Negeri Madura, Indonesia

Dear Editor,

We read with great interest the recent article by Valentino et al. (2026), which explored anxiety, depression, and perceived burden among patients with advanced cancer and their family caregivers. The study provides important longitudinal evidence showing that caregivers are strongly associated with psychological distress. These findings highlight the complex psychosocial dynamics within patient–caregiver dyads in advanced cancer.

While the study carefully addresses psychological and social contributions to distress, a spiritual–existential perspective may further enrich the interpretation of these findings. Patients with life-limiting illness often confront existential concerns such as loss of autonomy, fear of dependency, disrupted identity, and diminished sense of meaning. These experiences may intensify feelings of guilt toward caregivers and contribute to the perception of being a burden (Bernard et al. 2017). In this context, perceived burdensomeness may reflect deeper existential suffering rather than solely psychological distress.

Contemporary palliative care frameworks emphasize spirituality as a core dimension of holistic care alongside physical, psychological, and social domains. Spiritual well-being has been associated with improved quality of life and reduced anxiety and depression among patients with advanced illness (Balboni et al. 2013). Interventions such as meaning-centered psychotherapy and dignity therapy aim to restore meaning, dignity, and purpose in patients facing advanced disease and have shown benefits in reducing distress and improving spiritual well-being (Shen et al. 2025).

From this perspective, the perception of being a burden may represent an expression of existential distress related to loss of identity and autonomy. Integrating spiritual assessment and meaning-oriented interventions into routine palliative care may therefore complement psychological approaches and provide more comprehensive support for both patients and caregivers. Future research building on Valentino et al.’s work may benefit from incorporating measures of spiritual well-being and existential distress to better understand their relationship with perceived burden over time.
